# Investigating the role of MRGPRC11 and capsaicin-sensitive afferent nerves in the anti-influenza effects exerted by SLIGRL-amide in murine airways

**DOI:** 10.1186/s12931-016-0378-8

**Published:** 2016-05-23

**Authors:** Amy Y. Chang, Tracy S. Mann, Peter K. McFawn, Liang Han, Xinzhong Dong, Peter J. Henry

**Affiliations:** School of Medicine and Pharmacology, University of Western Australia, Crawley, WA 6009 Australia; School of Anatomy, Physiology & Human Biology, University of Western Australia, Crawley, 6009 WA Australia; Howard Hughes Medical Institute, Johns Hopkins University School of Medicine, Baltimore, MD 21205 USA

**Keywords:** SLIGRL-amide, Influenza, Airway sensory nerves, Capsaicin, Mas-related G protein-coupled receptor C11, Neuropeptides, Mucin

## Abstract

**Background:**

The hexapeptide SLIGRL-amide activates protease-activated receptor-2 (PAR-2) and mas-related G protein-coupled receptor C11 (MRGPRC11), both of which are known to be expressed on populations of sensory nerves. SLIGRL-amide has recently been reported to inhibit influenza A (IAV) infection in mice independently of PAR-2 activation, however the explicit roles of MRGPRC11 and sensory nerves in this process are unknown. Thus, the principal aim of this study was to determine whether SLIGRL-amide-induced inhibition of influenza infection is mediated by MRGPRC11 and/or by capsaicin-sensitive sensory nerves.

**Methods:**

The inhibitory effect of SLIGRL-amide on IAV infection observed in control mice in vivo was compared to effects produced in mice that did not express MRGPRC11 (*mrgpr-cluster∆*^*−/−*^ mice) or had impaired sensory nerve function (induced by chronic pre-treatment with capsaicin). Complementary mechanistic studies using both in vivo and ex vivo approaches investigated whether the anti-IAV activity of SLIGRL-amide was (1) mimicked by either activators of MRGPRC11 (BAM8-22) or by activators (acute capsaicin) or selected mediators (substance P, CGRP) of sensory nerve function, or (2) suppressed by inhibitors of sensory nerve function (e.g. NK1 receptor antagonists).

**Results:**

SLIGRL-amide and BAM8-22 dose-dependently inhibited IAV infection in *mrgpr-cluster∆*^*−/−*^ mice that do not express MRGPRC11. In addition, SLIGRL-amide and BAM8-22 each inhibited IAV infection in capsaicin-pre-treated mice that lack functional sensory nerves. Furthermore, the anti-IAV activity of SLIGRL-amide was not mimicked by the sensory neuropeptides substance P or CGRP, nor blocked by either NK1 (L-703,606, RP67580) and CGRP receptor (CGRP8-37) antagonists. Direct stimulation of airway sensory nerves through acute exposure to the TRPV1 activator capsaicin also failed to mimic SLIGRL-amide-induced inhibition of IAV infectivity. The anti-IAV activity of SLIGRL-amide was mimicked by the purinoceptor agonist ATP, a direct activator of mucus secretion from airway epithelial cells. Additionally, both SLIGRL-amide and ATP stimulated mucus secretion and inhibited IAV infectivity in mouse isolated tracheal segments.

**Conclusions:**

SLIGRL-amide inhibits IAV infection independently of MRGPRC11 and independently of capsaicin-sensitive, neuropeptide-releasing sensory nerves, and its secretory action on epithelial cells warrants further investigation.

## Background

Worldwide, influenza A virus (IAV) infections are estimated to cause up to 5 million cases of severe illness, and between 250,000 and 500,000 deaths each year [1]. The potentially devastating impact of seasonal influenza epidemics is lessened through vaccination, which significantly reduces the rate of hospitalisation and death from influenza. Unfortunately, annual vaccination does not provide protection against irregular influenza pandemics caused by antigenically novel strains of IAV transmitted from other animal species. Thus, considerable effort has been directed towards preventing and treating IAV infection with antiviral agents including the neuraminidase inhibitors oseltamivir (Tamiflu) and zanamivir (Relenza) [2, 3]. Despite these efforts, our capacity to control influenza infection remains threatened due to the rapid emergence of resistance to oseltamivir and other conventional antiviral agents such as the adamantanes [4–6]. Hence, there is an urgent unmet need to identify new anti-viral drugs to provide a frontline defence to infection, especially in the event of an unpredictable but inevitable influenza pandemic.

Our laboratory has recently shown that the hexapeptidic sequence SLIGRL-amide (Ser-Leu-Ile-Gly-Arg-Leu-NH_2_) inhibits IAV infection in mice [7]. SLIGRL-amide is a well-established activator of protease-activated receptor-2 (PAR-2) [8]. However, SLIGRL-amide inhibits IAV infection independently of PAR-2 as anti-viral effects were preserved in PAR2 (−/−) mice [7]. SLIGRL-amide has also been reported to activate MRGPRC11, a subtype of MAS-related G protein-coupled receptor (MRGPR) [8–10]. Whether SLIGRL-amide inhibits IAV infection via MRGPRC11 is currently unknown.

The MRGPR family comprises approximately 40 members that are grouped into nine subfamilies (MRGPRA to –H, and –X), based on sequence similarities. Subfamilies A, B, C, and H exist only in rodents, whereas subfamily X is specific to primates including humans [10]. Mouse and rat MRGPRC have been found to exhibit high similarities with human MRGPRX1 in terms of expression pattern, sequence homology and binding profile [11–14]. For example, both rodent MRGPRC11 and human MRGPRX1 are activated by the agonist BAM8-22. The expression of these receptors is primarily restricted to the small-diameter nociceptive sensory neurons [11, 12, 15, 16].

Sensory nerves of the respiratory system innervate a range of important structures, including the airway epithelium, submucosal glands and airway smooth muscle [17]. The predominant subtype of airway sensory nerve is the vagal bronchopulmonary C-fibre, whose cell bodies are located within the jugular and nodose ganglia [18]. Jugular C-fibres are more likely to innervate the upper airways [19] and to express neuropeptides when activated [20]. Noxious substances such as the TRPV1 activator capsaicin can activate populations of sensory nerves and may promote the release of neuropeptides such as substance P and CGRP via an axonal reflex [21]. Released neuropeptides, via activation of their cognate neurokinin (NK) and CGRP receptors, can in turn induce a wide range of biological effects, such as increased cilia beating and mucus secretion [22]. Several components of mucus, including surfactants, secretory IgA, defensins and MUC5B mucin have been reported to exhibit anti-IAV activities [23].

Thus far, the molecular target mediating the anti-IAV effects of SLIGRL-amide is unknown. The current study tests the hypotheses that SLIGRL-amide inhibits IAV infection by activating MRGPRC11, and that the antiviral effects of SLIGRL-amide are mediated by neuropeptides, which stimulate the secretion of endogenous substances with anti-IAV activities from mucus-producing airway cells. If the hypotheses are correct, then SLIGRL-amide induced anti-IAV effects should be mimicked by activators of MRGPRC11 (such as BAM8-22) and by exogenous neuropeptides (such as substance P and CGRP), as well as by other stimuli that directly activate sensory nerves (TRPV1 activator capsaicin). Furthermore, the anti-IAV effects of SLIGRL-amide should be reduced in mice lacking MRGPRC11 (*Mrgpr-cluster∆*^*−/−*^ mice, [8, 24]) and by processes (capsaicin-induced desensitisation) or agents (neurokinin receptor antagonists) that suppress sensory nerve function. In the current study, the effect of these interventions on SLIGRL-amide-induced anti-IAV activity was tested using a combination of *in vivo* (airway inflammation in IAV-exposed mice) and novel *ex vivo* approaches (immunohistochemical staining for IAV in viable perfused tracheal explants; [7]). Utilisation of *ex vivo* approaches also facilitated preliminary investigations of the anti-IAV activity of SLIGRL-amide in human isolated airways.

## Methods

### Ethics statement

All in vivo animal studies were conducted with the approval of the University of Western Australia Animal Ethics Committee (approval number RA/3/100/1258) and strictly adhered to the guidelines stipulated in the “Australian code for the care and use of animals for scientific purposes 8th Edition 2013”, published by the National Health and Medical Research Council of Australia. In accordance with the National Health and Medical Research Council of Australia’s National Statement on Ethical Conduct in Human Research 2007, segments of human lung tissue were obtained with informed written consent from patients undergoing lobectomy at two West Australian hospitals, Sir Charles Gairdner Hospital and the Mount Hospital. These studies were specifically approved by the Sir Charles Gairdner Hospital Human Research Ethics Committee (approval number 2011–128), the Mount Hospital Ethics Committee (approval number EC71.1) and The University of Western Australia Human Ethics Office (approval numbers RA/4/1/7256 and RA/4/1/7220).

### Influenza virus

Mouse-adapted influenza A/PR/8/34 virus was propagated in the allantoic fluid of 9-day old embryonated chicken eggs (Altona Hatchery, Forrestfield, Australia) at 37 °C for 3 days, as described previously [25]. Viral infectivity was assessed using allantois-on-shell titration and quantitated via hemagglutination assay [26]. The TCID50 of the harvested allantoic fluid was 10^-5.8^/ml.

### In vivo infection of mice with IAV

Unless otherwise stated, male BALB/c mice (specified pathogen-free) aged 7 to 8 weeks (Animal Resource Centre, Murdoch, WA) were housed at the University of Western Australia Animal Care Unit under a 12 h light/dark cycle and received food and water *ad libitum*. The role of MGRPRC11 in SLIGRL-amide-induced anti-IAV activity was investigated using *Mrgpr-cluster∆*^*−/−*^ and wild-type mice, generated as previously described [24]. Groups of mice were lightly anaesthetised (methoxyflurane) and intranasally (i.n.) inoculated with a 20 μl solution containing (a) influenza A/PR/8/34 virus (1:800 dilution of stock IAV) alone, (b) IAV plus peptide (SLIGRL-amide, SLIGR-amide, BAM8-22) or (c) 1:800 dilution of allantoic fluid (vehicle). Mice were killed with an overdose of pentobarbitone (160 mg/kg i.p. injection) at day 4 post-inoculation for determination of IAV-induced inflammation using differential cell counting of leukocytes recovered from bronchoalveolar lavage (BAL) fluid [7].

### Bronchoalveolar lavage

BAL was performed by intratracheal instillation of 2.5 ml of cold phosphate-buffered saline (PBS) pH 7.4, in 0.5 ml volumes via a tracheal cannula. After each instillation, BAL fluid was recovered, pooled and centrifuged at 400 × *g* for 5 mins at 4 °C. Supernatant was removed and the cell pellet resuspended in PBS + 1.0 % bovine serum albumin. Total cell counts and viability were determined by use of a haemocytometer and 0.4 % trypan blue exclusion. Cytospin preparations of each cell sample were stained with DIFF-Quik (Thermo Fisher Scientific, Waltham, MA), and differential cell counts of macrophages, neutrophils, eosinophils and lymphocytes were determined by counting 400 cells under a light microscope using standard morphological criteria.

### Capsaicin-induced attenuation of sensory nerve function in mice

Administration of multiple subcutaneous injections of capsaicin to anaesthetised mice is an effective means of impairing sensory nerve function [27–30]. On day one, mice were anaesthetized (130 mg/kg ketamine and 13 mg/kg xylazine, i.p.) and administered the bronchodilators terbutaline (0.1 mg/kg, i.p.) and theophylline (10 mg/kg, s.c.). Twenty mins later, mice were injected subcutaneously with capsaicin (25 mg/kg, s.c.) in the neck region between the shoulder blades. On days two and three, the procedure was repeated with an increased dose of capsaicin (50 mg/kg, s.c) (capsaicin-pretreated). Mice were left for 10 days before being intranasally inoculated with IAV ± SLIGRL-amide, as described above. Control mice received injections of vehicle (1:1:8 vol/vol/vol of ethanol/Tween 80/saline) (sham-pretreated). The protocol is shown in Fig. 3a. The effectiveness of the capsaicin desensitization protocol was determined in isometric tension recording studies by examining the capsaicin-induced relaxation responses in mouse isolated tracheal smooth muscle preparations.

### Isometric tension recording studies

As previously demonstrated in our laboratories [31, 32] isometric tension recording studies can be used to determine the functionality of the sensory nerves within the mouse trachea. Groups of mice exposed to either capsaicin or saline (sham) mice were euthanased using pentobarbitone (160 mg/kg, i.p.). Tracheal segments were excised and suspended under a resting tension of 0.2 g in organ baths containing 20 ml of Krebs bicarbonate solution (117 mM NaCl, 5.36 mM KCl, 25 mM NaHCO_3_, 1.03 mM KH_2_PO_4_, 0.57 mM MgSO_4_.7H_2_O, 2.5 mM CaCl_2_, 11.1 mM D-glucose) maintained at 37 °C and bubbled continuously with 5 % CO_2_ and 95 % O_2_. Changes in tension were recorded via a 751mT miniTOBs organ bath system (DMT, Aarhus, Denmark) connected to a Powerlab system (ADInstruments Pty Ltd., Castle Hill, Australia). Tracheal preparations were allowed to equilibrate for 30 mins and the viability of the tracheal smooth muscle was determined by cumulative exposure to a submaximal (0.2 μM) and maximal (10 μM) dose of carbachol. Tracheal segments were then repeatedly washed and allowed to rest for 20 mins. Preparations were precontracted using 1 μM carbachol and upon reaching a plateau level of tension were exposed to a single 20 μM bolus dose of capsaicin, and tension recordings continued until the peak response was obtained.

### Ex vivo infection of mouse isolated tracheal segments with IAV

An airway explant perfusion system developed in our laboratories was used as a means of investigating the effects of selected peptides (SLIGRL-amide, BAM8-22, substance P, CGRP) on the capacity of IAV to infect and replicate in murine isolated airways, as previously described [7]. Briefly, tracheae harvested from untreated mice (male BALB/c mice at 7 to 8 weeks of age) were mounted onto an 18G blunted needle and perfused for at least half an hour at 37 °C with complete RPMI (cRPMI) medium at the rate of 100 μL/min. Complete RPMI (cRPMI) medium consisted of RPMI 1640 medium (Gibco, Life Technologies-Thermo Fisher Scientific Inc, MA, USA) supplemented with 20 mM HEPES, 2 mM GlutaMAX™, 2.0 μg/mL amphotericin B, 20 μg/mL gentamycin, and 1 % foetal calf serum (FCS). Following this equilibration period, the perfusion was stopped and the tracheal lumen gently flushed first with 0.5 ml of sterile saline, and then air, before being exposed to IAV (1/800 dilution of stock) ± peptides for 15 mins, without perfusion. The lumen of the airway was then washed with 0.5 mL of sterile saline to remove unattached IAV and peptides, and perfused with cRPMI (100 μL/min at 37 °C) for 48 h. Airway segments were removed from the needle and fixed in 2 % paraformaldehyde (containing 0.2 % picric acid) in PBS (pH 7.4), for 48 h at 4 °C. Tissues were dehydrated (starting from 50 % ethanol) and processed to paraffin wax on a standard 15 h cycle in a Leica ASP200S automated tissue processor (Wetzlar, Germany).

### Isolated airways from human lung

The anti-IAV activity of SLIGRL-amide was also examined in small airway segments obtained from humans. Macroscopically normal samples of lung were obtained from patients undergoing lung surgery, usually for lung cancer. Segments of small airways (1–2 mm internal diameter) were dissected free from surrounding lung tissue and mounted onto the airway explant perfusion system. Perfused airways were exposed to IAV ± SLIGRL-amide, as outlined above for mouse studies.

### Immunohistochemistry and imaging

The presence of immunoreactive IAV in isolated trachea of IAV-exposed mice was visualised using standard immunohistochemical procedures, as previously reported [7]. Briefly, 5 μM wax sections were dewaxed, rehydrated and permeablized with 1 % Triton X-100 for 15 mins. Tissues were then blocked with 20 % normal rabbit serum or 3 % fish skin gelatin for 1 h and further exposed to avidin/biotin blocking as per manufacturer’s instructions (Avidin/Biotin Blocking Kit; Vector Laboratories, Burlingame, CA). Goat anti-influenza A polyclonal antibody, 1/1000 dilution (5 μg/ml) (Millipore Corporation, Billerica, MA); mouse anti-influenza A monoclonal antibody, 1/500 dilution (14.5 μg/ml) (Abcam, Cambridge, ENG); normal goat IgG isotype control or normal mouse serum were applied to sections and incubated overnight at 4 °C. After overnight incubation, sections were thoroughly washed in Tris-buffered saline (TBS) for one hour and endogenous peroxidase was quenched with 0.3 % H_2_O_2_ in TBS for 15 mins. The sections were then exposed to biotinylated rabbit anti-goat secondary IgG, 1/200 dilution (Vector Elite ABC kit; Vector Laboratories) or biotinylated rabbit anti-mouse IgG F(ab')2, 1/200 dilution (3.65 μg/ml) (Dako, Denmark) for 45 mins, followed by avidin-biotin-horseradish-peroxidase complex (Vector Elite ABC kit; Vector Laboratories) for 45 mins. The bound complex was visualised with diaminobenzidine (DAB, 0.4 mg/ml). Sections were counterstained with Mayers haematoxylin and blued with Scotts Tap Water Substitute, washed and dehydrated through graded alcohols to xylene, then coverslipped with Depex mounting medium. Digital images were acquired on an Aperio ScanScope XT digital slide scanner (Leica (Aperio) Technologies, Vista, CA). Images represent at least three animals per treatment.

Visualisation of immunoreactive IAV in human isolated bronchioles was achieved following the same protocol as for mouse tissue (described above) but with the following modifications - the diluent for both the primary and secondary antibodies was 3 % fish skin gelatin (FSG) (Sigma-Aldrich, St. Louis, MO) in 50 mM Tris + 0.5 M NaCl + 0.01 % Triton-X-100 . The blocking solution was 0.3 M glycine in 3 % FSG, and the wash buffer (50 mM Tris + 0.5 M NaCl + 0.01 % Triton-X-100) contained 0.3 M glycine. Avidin/Biotin blocking was not performed. All antibody concentrations, incubation and washing times, and visualisation with DAB remained the same.

### Quantitative analysis of immunoreactive IAV in mouse tracheal epithelium

Digital images of sections stained for immunoreactive IAV (and visualised with diaminobenzidine), were generated using a ScanScope XT digital slide scanner (Leica (Aperio) Technologies, Vista, CA) and analysed using ImageScope v11.1.2. The Positive Pixel Count v9 algorithm (Aperio Technologies) was applied with input parameters of - colour saturation threshold (0.01), hue value (0.1) and intensity ranges for weak positive (175–220), positive (100–175) and strong positive (0–175) pixels. These parameters were applied to an algorithm able to quantify diaminobenzidine positive sites of IAV expression, which were stained dark brown, as “strong positive pixels”. The number of strong positive pixels (SPP) in the epithelium was counted by the algorithm and expressed as a percentage of the total pixels within the epithelial layer (%SPP). %SPP was calculated for each tracheal section and provided a means of quantifying changes in levels of IAV staining.

### Attenuation of sensory nerve function in mouse isolated tracheal segments

To investigate whether capsaicin-induced inhibition of sensory nerve function blocks the anti-IAV effects of SLIGRL-amide *in vitro*, mouse tracheas from naïve mice were connected to the airway explant perfusion system and then exposed to capsaicin (20 μM) for 10 mins. Capsaicin-exposed preparations were washed and perfused with cRPMI for a further 10 mins prior to a standard 15 min exposure to IAV ± SLIGRL-amide. Tracheal segments were washed and perfused for 48 h with cRPMI, and then processed to determine levels of immunohistochemical staining for IAV. Complementary isometric tension recording studies were performed to confirm that *in vitro* capsaicin pre-treatment inhibited TRPV1-mediated activation of airway sensory nerves.

### Mucin secretion studies

The capacity of selected compounds to stimulate mucin release from epithelial stores of mucin was determined using the airway explant perfusion system. Following a 24 h period of perfusion with cRPMI, mouse isolated tracheal segments were exposed for 15 mins to SLIGRL-amide (200 μM), BAM8-22 (25 μM), substance P (1 μM), CGRP (1 μM) or to the known mucin secretagogue ATP (100 μM), or to saline. Tissues were then fixed in 2 % paraformaldehyde (containing 0.2 % picric acid) in PBS (pH 7.4), for 48 h at 4 °C, processed to paraffin wax and 5 μm sections stained with Alcian Blue-Periodic acid-Schiff’s reagent (AB-PAS) to demonstrate the presence of mucins (the chief glycoprotein constituent of mucus) in the epithelial layer. The chosen concentration and period of exposure to ATP were based on those employed in mucin release studies conducted in mice [33, 34].

### Quantitative analysis of AB-PAS staining in mouse tracheal epithelium

Digital images of AB-PAS stained sections were generated using a ScanScope XT digital slide scanner (Leica (Aperio) Technologies, Vista, CA) and analysed using ImageScope v11.1.2 and the Positive Pixel Count v9 algorithm (Aperio Technologies). Input parameters (color saturation threshold (0.04), hue value (0.1) and intensity ranges for weak positive (188–238), positive (176–188) and strong positive (0–176) pixels) were defined to generate an algorithm able to quantify AB-PAS-positive stores of mucin stained bright pink (as “strong positive pixels”). The number of strong positive pixels (SPP) in the epithelium was counted by the algorithm and expressed as a percentage of the total pixels within the epithelial layer (%SPP). %SPP was calculated for each tracheal section and provided a means of quantifying changes in levels of mucin staining.

### Materials

SLIGRL-amide, BAM8-22, SLIGR-amide and CGRP8-37 were supplied by Auspep (Melbourne, Australia). Stock solutions were prepared in sterile high-purity water with subsequent dilutions made in sterile saline. The concentration, purity, and composition of the peptides were determined by high-performance liquid chromatography, mass spectrometry, and quantitative amino acid analysis. Carbachol (carbamylcholine chloride), ATP, indomethacin, atropine, propranolol, terbutaline, theophylline and L-703,606 were purchased from Sigma–Aldrich (St. Louis, MO), whilst RP-67580 ((3aR,7aR)-Octahydro-2-[1-imino-2-(2-methoxy-phenyl)ethyl]-7,7-diphenyl-4H-isoindol), substance P, CGRP and L-733,060 were obtained from Tocris Bioscience (Ellisville, MO). Sodium pentobarbitone was supplied by Virbac Australia (Peakhurst, NSW), methoxyflurane by Medical Developments International Ltd (Springvale, Australia) and Schiff reagent by Australian Biostain (Vic, Australia).

### Statistical analysis

Unless otherwise stated, grouped data are expressed as mean ± SEM. Statistical comparisons between groups were made using one-way ANOVA with post-hoc testing (Holm procedure for all pairwise comparisons or Dunnett’s test for comparisons to single control) using *InVivoStat* software. Differences between groups were considered statistically significant at *p* <0.05.

## Results

### Effect of selected peptides on IAV infection in mice in vivo

**Fig. 1:**
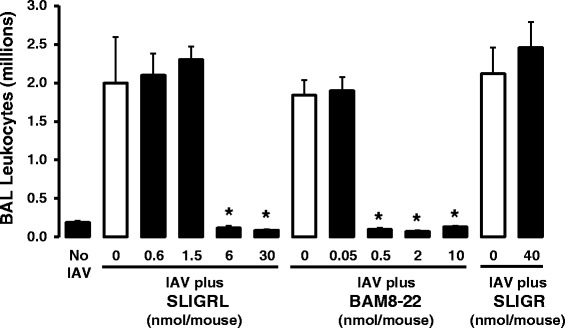
Measurement of relative anti-IAV activities of SLIGRL-amide, BAM8-22 and SLIGR in mice by counting leukocytes recovered from BAL fluid. Mice were anaesthetised and intranasally inoculated with IAV in the presence or absence of selected doses of peptides. Four days later, BAL leukocyte numbers were determined for each mouse. Shown are mean ± s.e.mean values from 4–10 mice/group. *, indicates *P* < 0.05 compared to respective IAV + saline control (one-way ANOVA)

As shown in Fig. 1, BAL fluid recovered from the lungs of sham-inoculated mice (No IAV) consistently contained approximately 0.15 million cells, of which 99 % were macrophages. In contrast, intranasal inoculation of IAV caused a marked influx of inflammatory cells into the lungs of mice, and by day 4 post-inoculation, the total BAL cell number was increased by approximately 10-fold, primarily due to a time-dependent influx of macrophages and neutrophils [7]. As expected, SLIGRL-amide (activator of PAR-2 and MRGPRC11; [8]) dose-dependently inhibited the IAV-induced increase in BAL leukocyte number (Fig. 1). BAM8-22, which activates MRGPRC11 but not PAR-2 [8]) also inhibited IAV-induced increases in BAL leukocyte number, and was approximately 10-times more potent than SLIGRL-amide (Fig. 1). In contrast, SLIGR-amide (activator of PAR-2 but not MRGPRC11; [8, 35]) did not inhibit IAV-induced increases in total BAL cell number at the highest dose tested (40 nmol/mouse) (Fig. 1). On face value, these data support the postulate that MRGPRC11 mediates the anti-IAV activities of SLGRL-amide and BAM8-22. However caution is required in this interpretation as SLIGRL-amide and BAM8-22 may not specifically activate MRGPRC11, and other receptors may be involved in mediating their anti-IAV effects.

### Anti-IAV activity of SLIGRL-amide and BAM8-22 in Mrgpr-cluster∆^−/−^ and wild-type mice

**Fig. 2:**
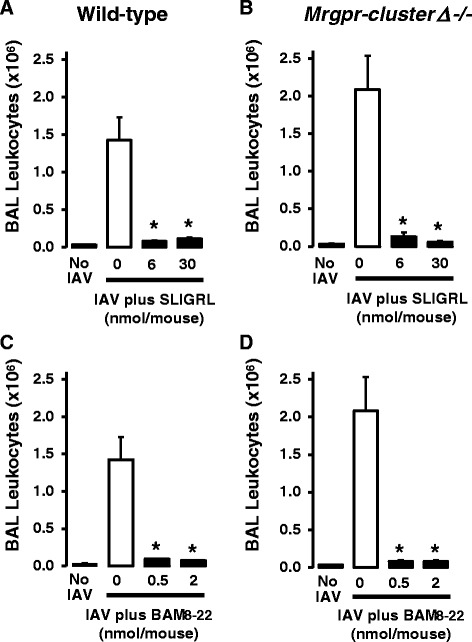
Measurement of anti-IAV activities of SLIGRL-amide and BAM8-22 in wild-type and *Mrgpr-cluster∆* ^*−/−*^ mice by counting BAL leukocytes. Wild-type (**a**, **c**) and *Mrgpr-cluster∆* ^*−/−*^ (**b**, **d**) mice were anaesthetised and intranasally inoculated with IAV and selected doses of SLIGRL-amide (**a**, **b**) or BAM8-22 (**c**, **d**). Four days later, BAL was performed on each mouse and the total numbers of leukocytes recovered was determined. Shown are mean ± s.e.mean of 3 mice per group. *, indicates *P* <0.05, compared to respective IAV + saline control (one-way ANOVA)

To definitively evaluate the role of MRGPRC11 in the anti-IAV activities of SLIGRL-amide and BAM8-22, studies were conducted using *Mrgpr-cluster∆*^*−/−*^ mice that do not express MRCPRC11 [8, 24]. Intranasal inoculation of mice (*Mrgpr-cluster∆*^*−/−*^ and wild-type) with IAV (in the absence of peptides) was associated with a marked increase in total BAL cell number on day 4 post-inoculation (Fig. 2). As expected, both SLIGRL-amide (6 and 30 nmol/mouse) and BAM8-22 (0.5 and 2 nmol/mouse) inhibited the IAV-induced increase in total BAL cell number in wild-type mice [24] (Fig. 2a and 2c). However, contrary to the hypothesis that MRGPRC11 mediates the anti-IAV effects of SLIGRL-amide and BAM8-22, doses of these peptides that inhibited IAV-induced increases in total BAL cell number in BALB/c mice (Fig. 1a) and wild-type mice (Fig. 2), also exhibited anti-IAV activity in *Mrgpr-cluster∆*^*−/−*^ mice (Fig. 2b and 2d). These findings provide strong evidence that the anti-IAV activities of either SLIGRL-amide or BAM8-22 are not mediated by MRGPRC11.

### Role of sensory nerves in SLIGRL-amide-induced anti-IAV activity

Further studies were completed to evaluate the possibility that the anti-IAV activity of SLIGRL-amide was mediated by capsaicin-sensitive, neuropeptide-releasing airway sensory nerves. In these studies, the activity of SLIGRL-amide was investigated in mice in which sensory nerve function had been attenuated by either (1) chronic exposure to capsaicin or (2) administration of a neurokinin receptor antagonist.

**Fig. 3:**
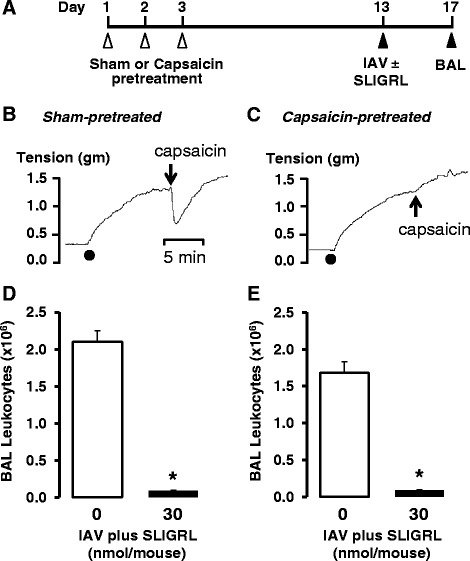
Effect of sensory nerve dysfunction caused by *in vivo* capsaicin treatment on the anti-IAV activity of SLIGRL-amide in mice. **a** Protocol for capsaicin-induced dysfunction of sensory nerves. On 3 consecutive days, groups of mice were anaesthetised and injected subcutaneously with capsaicin or vehicle (Sham). Ten days later, mice were anaesthetised and intranasally inoculated with IAV in the presence or absence of SLIGRL-amide. A further four days later, BAL was performed on each euthanased mouse and the total numbers of leukocytes recovered was determined. **b** & **c** Isometric tension recordings performed on day 13 show that capsaicin pretreatment (using the protocol in **a**) abolishes the characteristic relaxant response to capsaicin (10 μM), indicative of sensory nerve dysfunction. **d** & **e** Total BAL leukocyte numbers recovered from IAV-inoculated mice that had been sham-pretreated (**d**) or pretreated with capsaicin to induce sensory nerve dysfunction. Shown are mean ± s.e.mean of 3–7 mice per group. *, indicates *P* <0.05, compared to respective IAV + saline control (one-way ANOVA)

Repeated daily exposure to capsaicin has previously been reported to cause chemical ablation of sensory nerves in mice [27, 29, 36–38]. In our studies, anaesthetized mice were exposed to capsaicin on 3 consecutive days (see Fig. 3a for protocol), and confirmation of the effectiveness of this *in vivo* protocol to induce airway sensory nerve dysfunction was obtained from isometric tension recording experiments (Fig. 3b and 3c, lower panels). Tracheal smooth muscle segments from capsaicin-pre-treated mice did not relax in response to capsaicin, compared to the large relaxation response obtained in sham-pre-treated (no capsaicin) mice (Fig. 3b and 3c), consistent with sensory nerve dysfunction.

Inoculation with IAV on day 13 of the protocol produced similar increases in BAL leukocytes in sham and capsaicin-pretreated mice (Fig. 3d and 3e). Moreover, IAV-induced increases in BAL leukocytes were inhibited by SLIGRL-amide in both sham (Fig. 3d) and capsaicin-pretreated mice (Fig. 3e). These findings do not support a role for classic TRPV1-expressing, sensory C-fibres in SLIGRL-amide-induced inhibition of IAV infectivity.

**Fig. 4:**
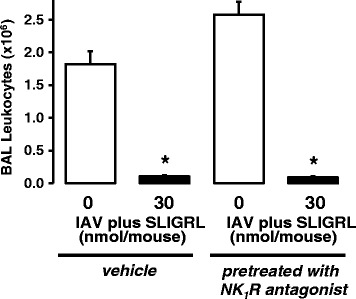
Effect of *in vivo* administration of an NK1 receptor antagonist on the anti-IAV activity of SLIGRL-amide in mice. Groups of mice were injected with L-703,606 (8.5 mg/kg, i.p.) or vehicle (control), and 30 mins later intranasally inoculated with IAV in the presence or absence (saline) of SLIGRL-amide. A further four days later, BAL was performed on each euthanased mouse and the total numbers of leukocytes recovered was determined, as shown (mean ± s.e.mean of 4–5 mice per group). *, indicates *P* <0.05 compared to respective IAV + saline control (one-way ANOVA)

Some populations of activated airway sensory nerves release neuropeptides such as the tachykinin substance P, which transmits responses via activation of neurokinin (NK) receptors. Consistent with this, NK1 receptor antagonists inhibit substance P-induced effects on microvascular leakage, mucus secretion and bronchomotor tone in the airways (see [39]). In the current study, administration of the NK1 receptor antagonist L-703,606 (8.5 mg/kg; [40, 41]) did not block the capacity of SLIGRL-amide to inhibit IAV-induced increases in total BAL cell number in mice (Fig. 4), indicating that the actions of SLIGRL-amide were not mediated via activation of the sensory nerve – substance P – NK1 receptor axis.

### Effect of SLIGRL-amide and BAM8-22 on IAV infectivity in mouse isolated trachea ex vivo

**Fig. 5:**
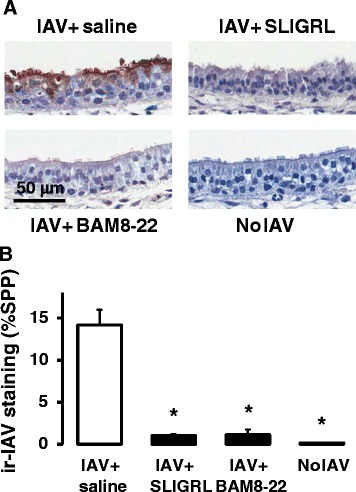
Semiquantitative measurement of anti-IAV activities of SLIGRL-amide and BAM8-22 in mouse isolated tracheal segments by immunohistochemical staining of IAV nucleoprotein. **a** Immunohistochemical staining for IAV nucleoprotein in mouse tracheal sections. Mouse tracheal segments were mounted onto an airway explant perfusion system and incubated for 15 mins with IAV in the presence or absence of SLIGRL-amide or BAM8-22. Some tracheal segments were exposed to dilute allantoic fluid instead of IAV (No IAV). After 48 h of perfusion with media, segments were fixed, paraffin-embedded, sectioned and immunohistochemically stained for IAV. Shown are representative images from at least 3 experiments. Bar = 50 μm. **b** Levels of staining for immunoreactive-IAV in the epithelium of perfused trachea exposed to IAV in the absence (*n* = 29 trachea) or presence of SLIGRL-amide (*n* = 8 trachea) or BAM8-22 (*n* = 4 trachea). Some tracheal segments were exposed to dilute allantoic fluid instead of IAV (No IAV; *n* = 3 trachea). Shown are mean levels of immunoreactive IAV staining (and standard error of the mean), expressed as % of strong positive pixels, as outlined in the Methods section. *, indicates *P* <0.05, compared to IAV + saline control (one-way ANOVA)

Our laboratories have previously demonstrated that the antiviral activity of SLIGRL-amide observed in intact mice can be replicated in mouse isolated trachea *ex vivo* [7]. The novel *ex vivo* perfused tracheal system preserves tissue viability, architecture and microenvironment of the airways, and enables the study of the cellular mechanisms through which SLIGRL-amide inhibits IAV virus infection. Furthermore, this *ex vivo* perfusion system can be adapted to airways of other non-murine species, including human airways. In *ex vivo* studies, the levels of immunoreactive staining for IAV within the airway epithelium is the primary endpoint used to indicate IAV infectivity [7]. Extensive immunoreactive staining for IAV is indicative that the virus has proceeded through the sequential processes of viral attachment, endocytosis and replication. In the current study, a brief 15 min exposure of mouse isolated trachea to IAV resulted in the subsequent propagation of IAV within the epithelium over the ensuing 48 h, as revealed by the presence of high levels of staining for immunoreactive IAV (Fig. 5a and 5b). In stark contrast, mouse tracheal segments exposed to SLIGRL-amide and BAM8-22 during the 15 min exposure period to IAV revealed negligible levels of immunohistochemical staining for IAV (Fig. 5a and 5b), indicating that IAV infectivity had been impaired by these peptides. Thus, as well as inhibiting IAV-induced increases in total BAL cell number *in vivo* (Fig. 1), SLIGRL-amide and BAM8-22, also inhibited IAV infectivity in mouse isolated tracheal segments (Fig. 5).

### Effect of sensory nerve activation on IAV infectivity

**Fig. 6:**
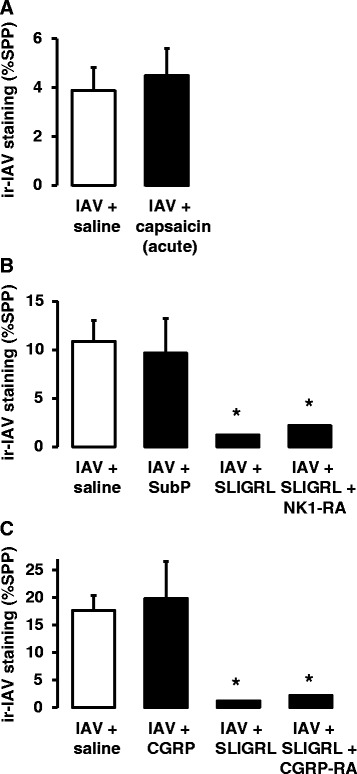
Effect of neuropeptide receptor antagonists on the anti-IAV activities of SLIGRL-amide in mouse isolated tracheal segments by immunohistochemical staining of IAV nucleoprotein. Levels of staining for immunoreactive-IAV in the epithelium of perfused trachea exposed to IAV in the absence or presence of single acute dose of (**a**) capsaicin (10 μM), (**b**) substance P (1 μM) or (**c**) CGRP (1 μM) (*n* = 3-15 trachea). Also shown are the effects produced by an NK1 receptor antagonist (20 μM RP67580; **b**) and a CGRP receptor antagonist (10 μM CGRP8-37; **c**) on the anti-IAV activity of SLIGRL-amide. Shown are mean levels of immunoreactive IAV staining (and standard error of the mean), expressed as % of strong positive pixels, as outlined in the Methods section. *, indicates *P* <0.05, compared to respective IAV + saline control (one-way ANOVA)

Consistent with *in vivo* studies, the evidence obtained from studies using isolated mouse tracheal segments did not support the postulate that the anti-IAV effects of SLIGRL-amide were mediated by activation of capsaicin-sensitive, neuropeptide-releasing sensory nerves. For example, the acute and direct activation of sensory nerves caused by a single 15 min exposure to capsaicin failed to inhibit IAV infectivity (Fig. 6a). Similarly, the exogenous application of selected sensory neuropeptides known to be released locally from activated sensory nerves (substance P or CGRP at 1 μM), did not mimic the anti-IAV effects of SLIGRL-amide (Fig. 6b and 6c). Furthermore, neither an NK1 receptor antagonist (RP67580, 20 μM) nor a CGRP receptor antagonist (CGRP8-37, 10 μM) blocked SLIGRL-amide-induced inhibition of IAV infectivity in mouse tracheal segments (Fig. 6b and 6c).

### Effect of SLIGRL-amide and ATP on release of epithelial mucin stores from mouse isolated tracheal segments

**Fig. 7:**
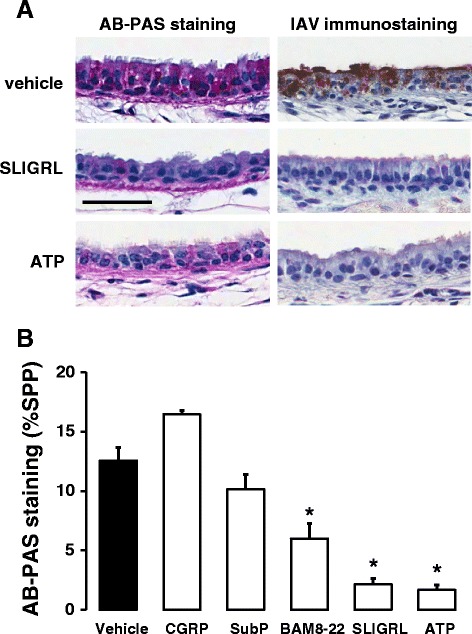
Comparative effects of SLIGRL-amide and ATP on promoting mucin release and inhibiting IAV infectivity in mouse isolated tracheal segments. **a** Effect of SLIGRL-amide and the purinoceptor agonist ATP on levels of AB-PAS (left-hand panels) and IAV (right-hand panels) staining in sections of mouse trachea. In AB-PAS experiments, mouse isolated tracheal segments were mounted onto the explant perfusion system and pre-perfused for 24 h, and then incubated for 15 min with saline, SLIGRL-amide or ATP. Segments were fixed, paraffin-embedded, sectioned and stained with AB-PAS. In IAV immunohistochemical staining experiments, tracheal segments were mounted onto the explant perfusion system and incubated for 15 min with IAV in the presence of absence of saline, SLIGRL-amide or ATP. After two days of perfusion with media, segments were fixed, paraffin-embedded, sectioned and immunostained for IAV. Shown are representative images from at least 3 experiments. Bar = 50 μm. **b** Residual levels of AB-PAS staining, expressed as %SPP (strong positive pixels), within the epithelium of mouse isolated tracheal segments following a 15 min exposure to saline (control, white fill) or selected compounds (black fill) (*n* = 4-8 tracheal segments per group). Luminal exposure to SLIGRL-amide, BAM8-22 and ATP, but not CGRP or substance P (SubP), stimulated a reduction in epithelial AB-PAS staining, consistent with mucin secretion. *, *P* <0.05, compared to vehicle (one-way ANOVA, Dunnett’s posthoc test)

Additional experiments were conducted to investigate the possibility that SLIGRL-amide stimulates the release of substances with anti-IAV activity directly from the airway epithelium. Consistent with this, exposure of tracheal segments to SLIGRL-amide was associated with a marked reduction in the levels of AB-PAS staining within the tracheal epithelium, indicative of mucin release (left-hand panels of Fig. 7a and 7b). Levels of AB-PAS staining were also reduced by the established secretagogue ATP and by BAM8-22, but not by either substance P or CGRP (Fig. 7b). Of particular interest, ATP also inhibited IAV infectivity in mouse isolated tracheal segments (right hand panels of Fig. 7a), similar to the effect produced by SLIGRL-amide.

### Effect of SLIGRL-amide on IAV infectivity in airways of human isolated airways

**Fig. 8:**
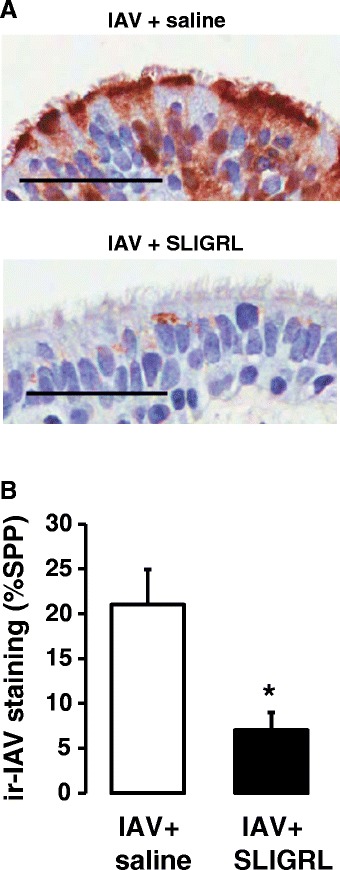
Semiquantitative measurement of anti-IAV activities of SLIGRL-amide in human isolated bronchiolar segments by immunohistochemical staining of IAV nucleoprotein. **a** Immunohistochemical staining of IAV in sections of human isolated bronchiolar segments exposed to IAV in the absence (upper image) or presence of SLIGRL-amide (lower image), for 15 min. After 48 h of perfusion with media, segments were fixed, paraffin-embedded, sectioned and immunohistochemically stained for IAV. Bar = 50 μm. **b** Levels of staining for immunoreactive IAV nucleoprotein in the bronchiolar epithelium of perfused human airways exposed to saline or SLIGRL-amide. Shown are representative images from at least 4–9 experiments using airways from different donors. *, indicates *P* <0.05, compared to IAV + saline control (one-way ANOVA)

As indicated above, an advantage of the *ex vivo* perfused airway system is that it can be readily adapted to enable the study of human airways. A 15 min exposure of human isolated bronchioles to IAV, followed by two days of perfusion with IAV-free cRPMI medium was associated with the development of high levels of staining for immunoreactive IAV within the epithelium, consistent with the replication of IAV (Fig. 8a). Co-incubation of IAV with SLIGRL-amide during the initial 15 min exposure period was associated with significantly suppressed the levels of epithelial IAV immunoreactivity in *ex vivo* segments of human bronchioles two days later (P = 0.05; Fig. 8b), consistent with findings obtained from *in vivo* and *ex vivo* studies using mice.

## Discussion

SLIGRL-amide activates murine PAR-2, and has been used for over twenty years to evaluate the role of PAR-2 activation in physiological and pathological processes [42–45]. SLIGRL-amide has also recently been shown to activate MRGPRC11, a G protein-coupled receptor expressed exclusively on sensory nerves [8], and to inhibit IAV infection in mice that lack PAR-2 [7]. The current study sought to determine whether the antiviral activity of SLIGRL-amide in mouse airways involved activation of MRGPRC11 and/or sensory nerves, and to determine whether SLIGRL-amide also inhibited IAV infectivity in human airways.

In the current study, IAV infectivity in intact mice and in mouse isolated tracheal segments was inhibited by BAM8-22, an established activator of MRGPRC11 [8, 13]. Indeed, the observed 10-fold greater anti-IAV potency of BAM8-22 compared to SLIGRL-amide in mice was consistent with an action on MRGPRC11 [8, 10]. However, both SLIGRL-amide and BAM8-22 were effective in preventing IAV infection in *Mrgpr-cluster∆*^*−/−*^ mice that do not express MRGPRC11 [8]. These latter studies conducted in *Mrgpr-cluster∆*^*−/−*^ mice provide compelling evidence that the anti-IAV activities of SLIGRL-amide and BAM8-22 were mediated independently of MRGPRC11. Additional pharmacologic studies were then undertaken to determine whether the anti-IAV properties of SLIGRL-amide and BAM8-22 involved activation of a population of capsaicin-sensitive, peptidergic airway sensory nerves.

We hypothesised that if SLIGRL-amide inhibits IAV infection via activation of capsaicin-sensitive sensory nerves, then the anti-IAV activity of SLIGRL-amide should be suppressed following the chemical ablation of sensory nerves by repeated exposure of rodents to capsaicin [27, 29, 36–38]. However, the anti-IAV effects produced by SLIGRL-amide were preserved in mice that had been exposed to capsaicin on three consecutive days 10 days prior to inoculation with IAV, despite tracheal preparations obtained from these mice failing to produce a characteristic relaxation response to capsaicin [32]. Consistent with the findings of these *in vivo* studies, *in vitro* pre-treatment of mouse isolated tracheal segments to capsaicin rendered them unresponsive to subsequent challenges to capsaicin, but not to the anti-IAV actions of SLIGRL-amide. Furthermore, although NK1 receptors have previously been implicated in SLIGRL-amide-induced actions in mouse trachea [46] and small intestine [47], neither NK1 nor CGRP receptor antagonists blocked the anti-IAV activities of SLIGRL-amide, *in vivo* or *ex vivo*. Thus, our data do not support the explicit hypothesis that SLIGRL-amide inhibits IAV via activation of capsaicin-sensitive sensory nerves, or via the actions of the neuropeptides substance P or CGRP.

In a complementary series of experiments, we tested the postulate that if SLIGRL-amide inhibits IAV infection via activation of sensory nerves and local release of neuropeptides, then other agents that stimulate sensory nerves would mimic the anti-IAV actions of SLIGRL-amide. Capsaicin directly activates sensory nerve C-fibres via stimulation of the highly expressed TRPV1 calcium ion channel. However, exposing mouse isolated tracheal segments to capsaicin together with IAV did not mimic the anti-IAV effects produced by SLIGRL-amide. In these experiments, capsaicin was used at a concentration (10 μM) that induced large relaxation responses in mouse isolated tracheal preparations [32]. These responses were blocked by a TRPV1 antagonist (capsazepine) and an NK1 receptor antagonist (L-733060), consistent with sensory nerve activation [32]. In addition, if the anti-IAV effects of SLIGRL-amide were mediated via the actions of substance P and/or CGRP released from activated sensory nerves, then the exogenous application of these neuropeptides should also inhibit IAV infectivity. However, neither substance P nor CGRP, at concentrations known to induce maximal receptor activation, inhibited IAV infectivity in mouse isolated tracheal preparations. Together, these findings do not support the postulate that activation of capsaicin-sensitive, neuropeptide-releasing sensory nerves at the time of IAV exposure significantly inhibits IAV infectivity in the epithelium of mouse isolated trachea.

Sensory nerve C fibre function has been reported to influence the response of the host to respiratory tract insults including bacterial endotoxin, mycoplasma and environmental toxicants [27, 37, 38, 48–50], however little is known of the consequence of sensory nerve activation or sensory nerve dysfunction on influenza infection. In the current study, stimulation of sensory nerves with capsaicin during an initial period of IAV exposure did not prevent the subsequent propagation of IAV within the epithelium of mouse isolated airways. These findings indicate that local release of neuropeptides following sensory nerve activation does not markedly impact on the capacity of IAV to enter and replicate in airway epithelial cells.

The current study has established that SLIGRL-amide inhibits IAV infectivity *in vivo* and *ex vivo* via mechanisms that are independent of MRGPRC11 and of capsaicin-sensitive sensory nerves, and also independent of the sensory neuropeptides substance P and CGRP and their receptors. However, it is important to note that vagal bronchopulmonary C-fibers in the mouse are heterogeneous – a substantial fraction of C-fiber afferent nerves in the mouse respiratory system are capsaicin-insensitive [51], and that a fraction of capsaicin-sensitive C-fibers are non-peptidergic [20]. Thus, we cannot exclude the possibility that SLIGRL-amide may exert its antiviral activities through actions on sub-populations of capsaicin-insensitive, nonpeptidergic neurons. Nevertheless, the current investigation builds on our previous studies that demonstrate SLIGRL-amide does not inhibit IAV infectivity via PAR-2, by direct antiviral or by immunomodulatory processes [7]. Of particular interest, the current study has shown that the anti-IAV effects of SLIGRL-amide observed in murine airways extend to human airways, although further studies are required to identify its specific molecular target.

SLIGRL-amide and related peptides have been reported to directly stimulate secretory pathways in the airways [46, 52–54]. Importantly, components of airway secretions, including the mucin MUC5AC exert protective roles against influenza A virus [55]. Thus, the release of endogenous antiviral substances in mucus may contribute to the anti-IAV activity of SLIGRL-amide. In the current study, SLIGRL-amide promoted the release of intracellular mucins from mouse tracheal epithelium, as determined from microscopic imaging of AB-PAS-stained airway sections. The purinergic receptor agonist ATP, an established key secretagogue for airway epithelium [56–58], also promoted the release of intracellular mucins from mouse isolated tracheal epithelium, and moreover, inhibited IAV infectivity. These findings provide preliminary evidence that SLIGRL-amide may inhibit IAV infectivity by stimulating the secretion of endogenous substances with anti-IAV activity. Nevertheless, it remains to be determined whether the anti-IAV activity of SLIGRL-amide involves the secretion of sialic acid-rich mucins [55], or other anti-IAV molecules such as cationic host defence peptides (β-defensins, cathelicidins) [59] or phospholipids such as palmitoyl-oleoyl-phosphatidylglycerol (POPG) [60].

## Conclusion

The current study using a combination of *in vivo* and *ex vivo* approaches provides compelling evidence that the anti-IAV activity of SLIGRL-amide in murine airways occurs independently of the sensory nerve receptor MRGPRC11 and of neuropeptide release from TRPV1-expressing sensory nerves. Preliminary data show that both SLIGRL-amide and ATP release airway secretions and inhibit IAV infection, raising the possibility that SLIGRL-amide inhibits IAV infectivity via the release of antiviral substances from the airway epithelium. Additional release and intervention studies, particularly in human airways, will provide invaluable insight into the molecular target and cellular signalling processes through which SLIGRL-amide inhibits IAV infectivity.
